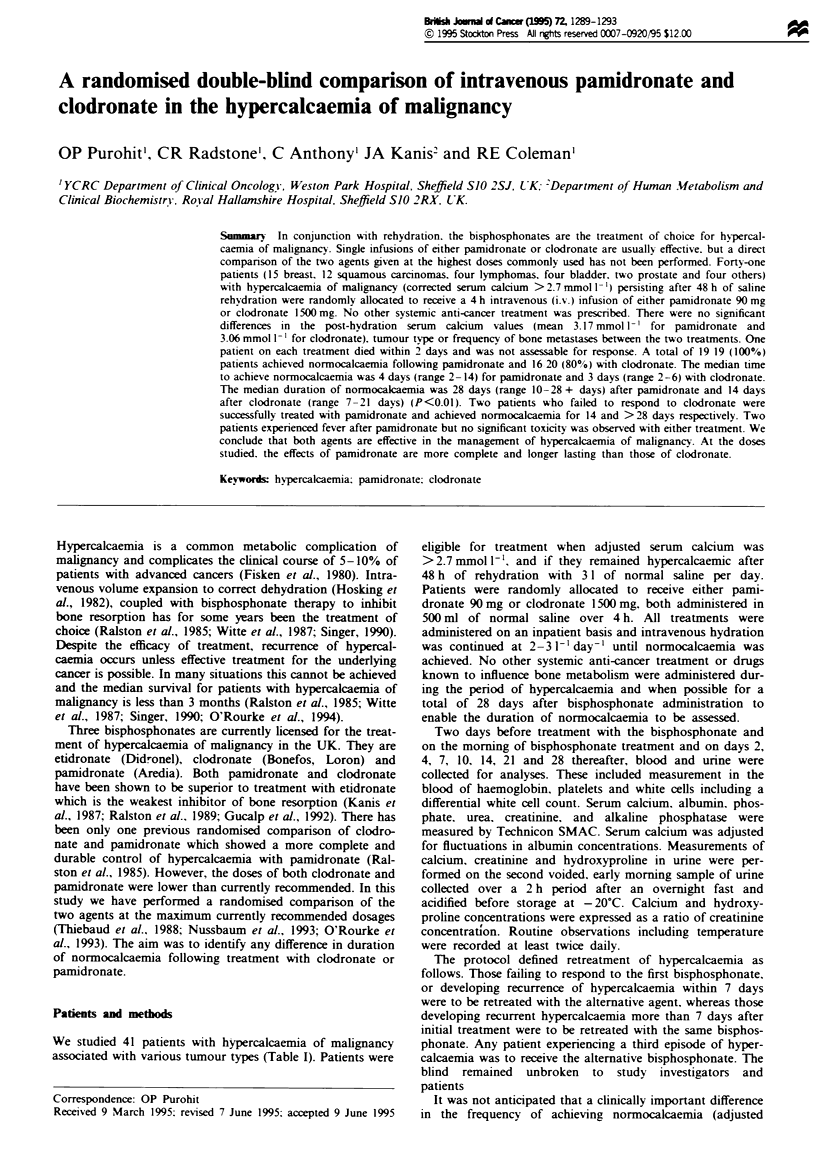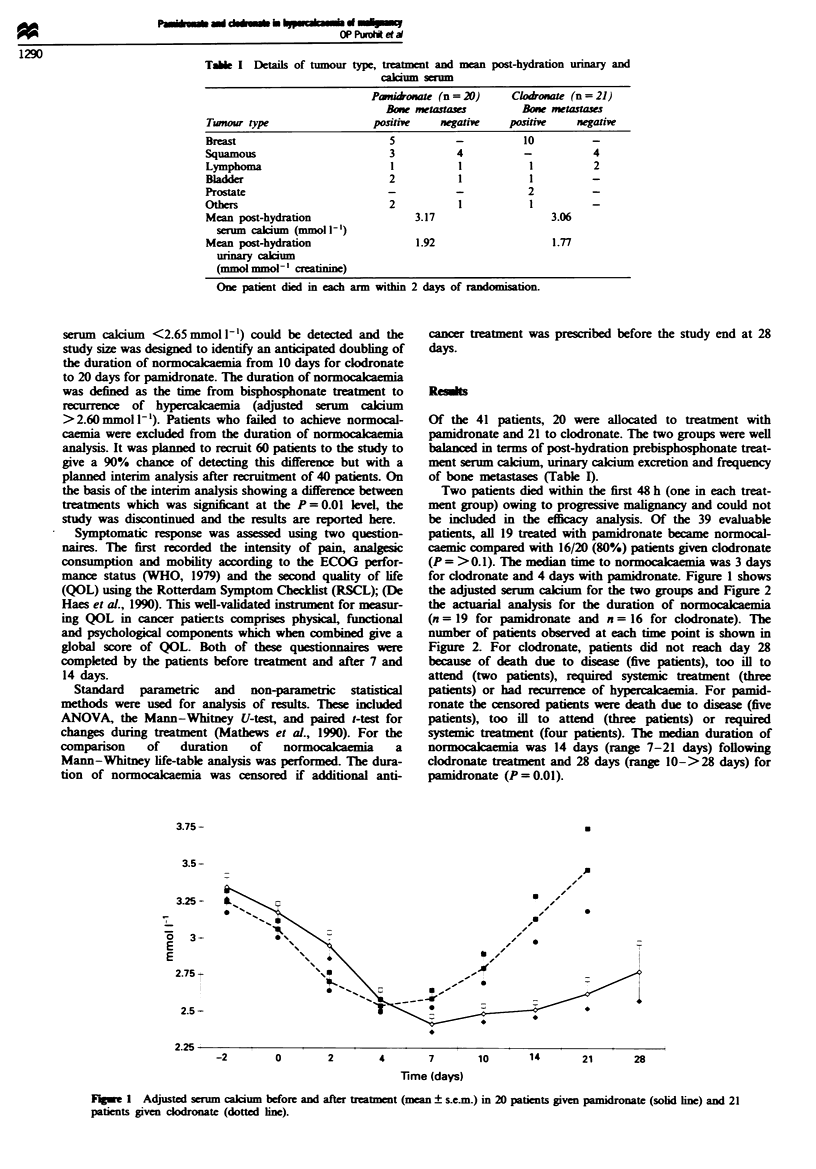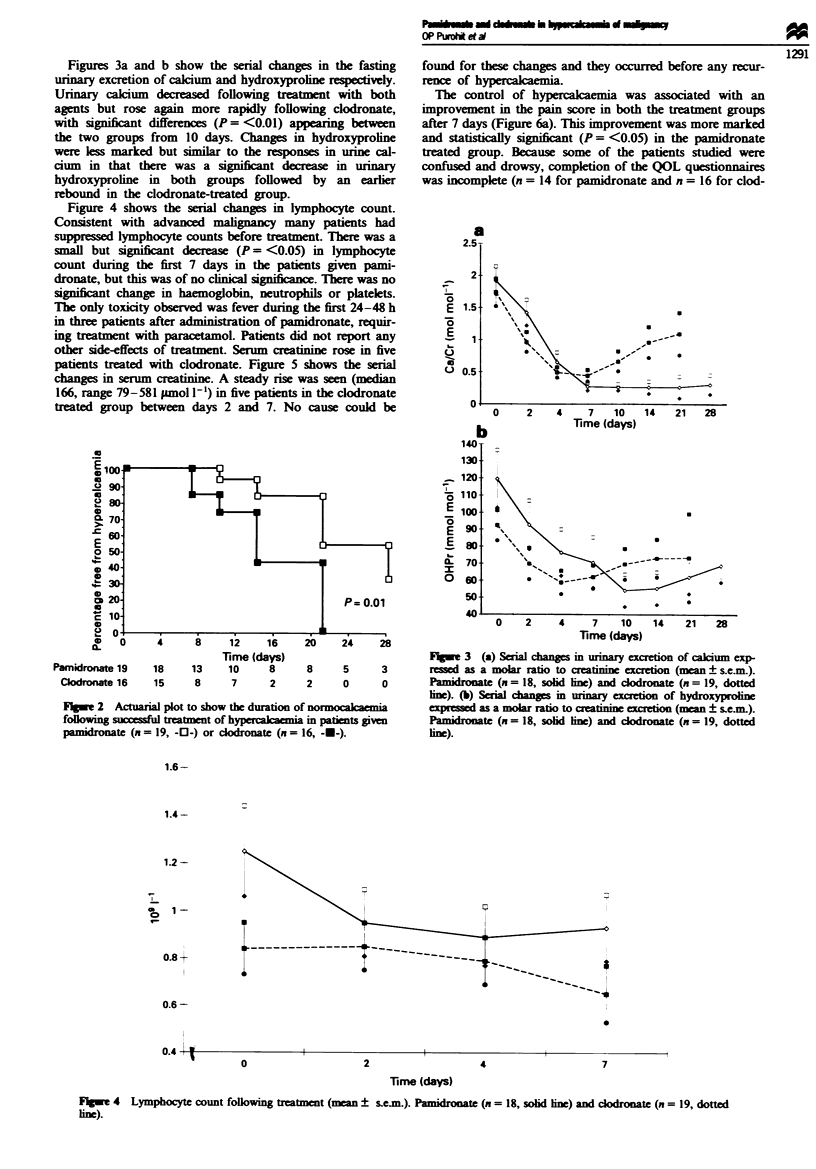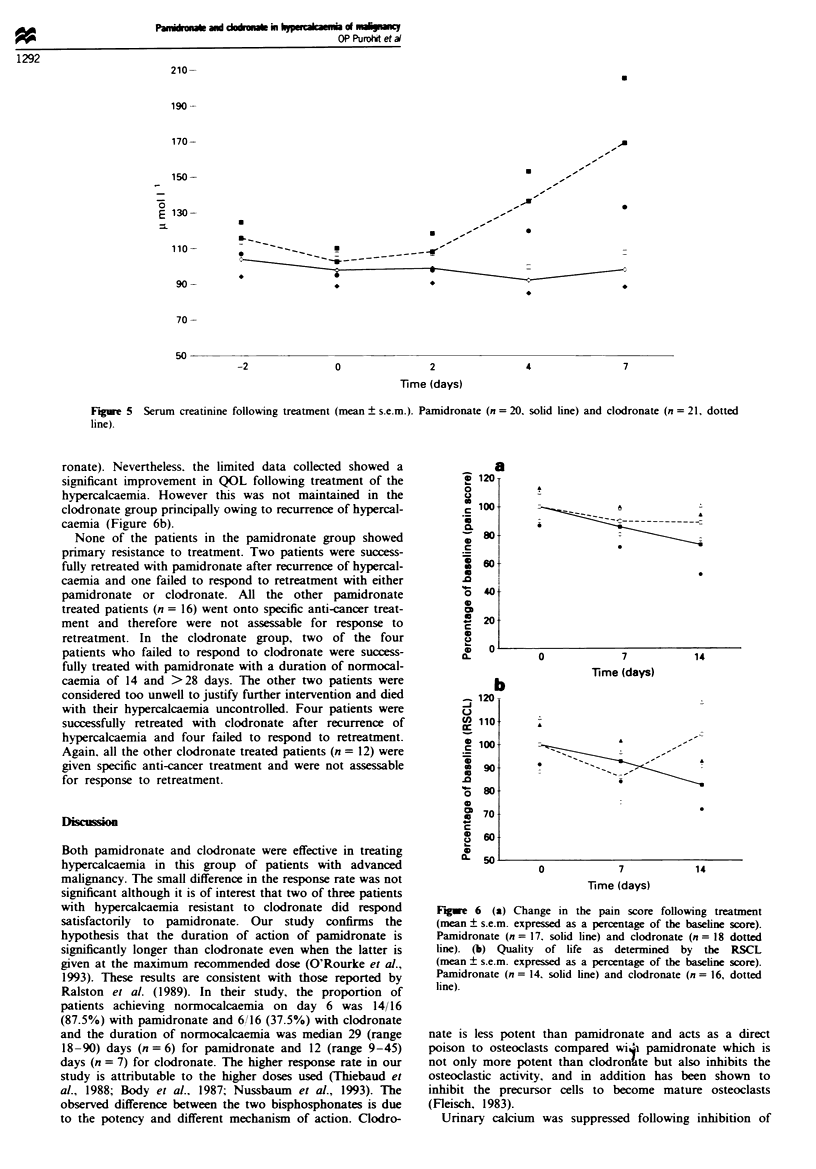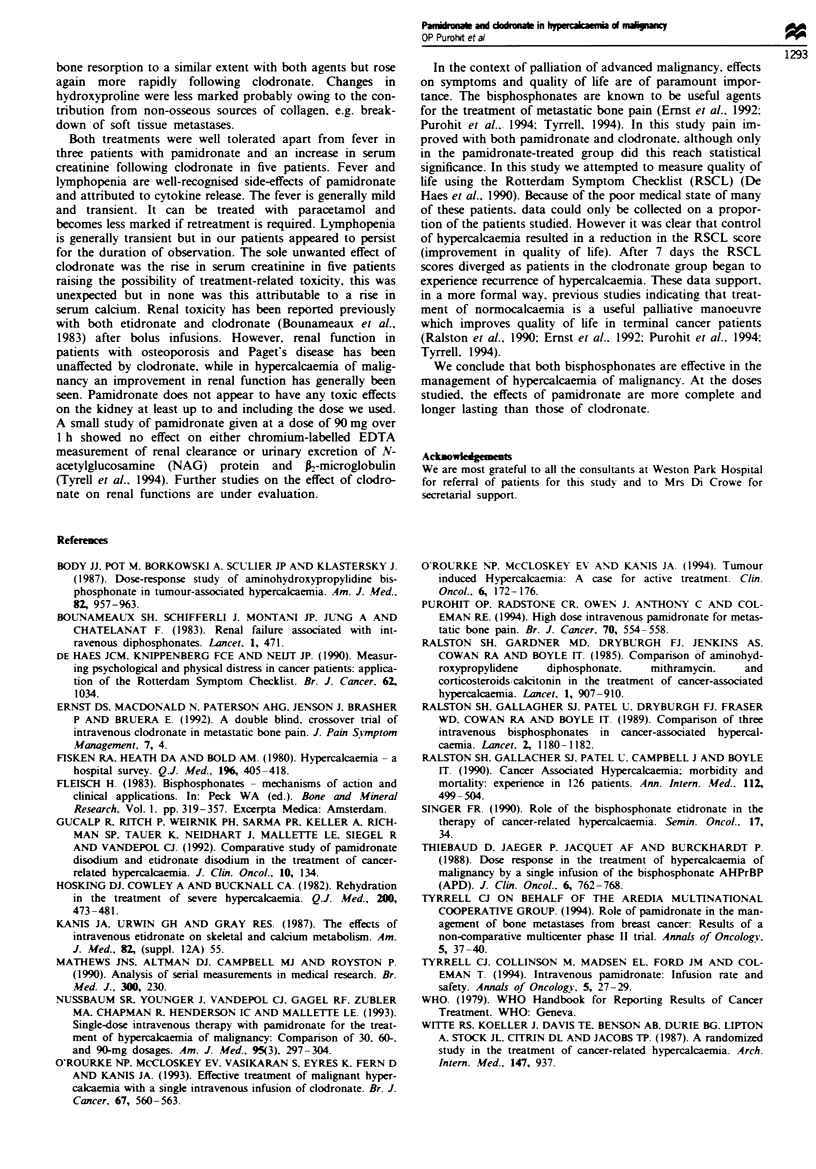# A randomised double-blind comparison of intravenous pamidronate and clodronate in the hypercalcaemia of malignancy.

**DOI:** 10.1038/bjc.1995.502

**Published:** 1995-11

**Authors:** O. P. Purohit, C. R. Radstone, C. Anthony, J. A. Kanis, R. E. Coleman

**Affiliations:** YCRC Department of Clinical Oncology, Weston Park Hospital, Sheffield, UK.

## Abstract

In conjunction with rehydration, the bisphosphonates are the treatment of choice for hypercalcaemia of malignancy. Single infusions of either pamidronate or clodronate are usually effective, but a direct comparison of the two agents given at the highest doses commonly used has not been performed. Forty-one patients (15 breast, 12 squamous carcinomas, four lymphomas, four bladder, two prostate and four others) with hypercalcaemia of malignancy (corrected serum calcium > 2.7 mmol l-1) persisting after 48 h of saline rehydration were randomly allocated to receive a 4 h intravenous (i.v.) infusion of either pamidronate 90 mg or clodronate 1500 mg. No other systemic anti-cancer treatment was prescribed. There were no significant differences in the post-hydration serum calcium values (mean 3.17 mmol l-1 for pamidronate and 3.06 mmol l-1 for clodronate), tumour type or frequency of bone metastases between the two treatments. One patient on each treatment died within 2 days and was not assessable for response. A total of 19/19 (100%) patients achieved normocalcaemia following pamidronate and 16/20 (80%) with clodronate. The median time to achieve normocalcaemia was 4 days (range 2-14) for pamidronate and 3 days (range 2-6) with clodronate. The median duration of normocalcaemia was 28 days (range 10-28+ days) after pamidronate and 14 days after clodronate (range 7-21 days) (P < 0.01). Two patients who failed to respond to clodronate were successfully treated with pamidronate and achieved normocalcaemia for 14 and > 28 days respectively. Two patients experienced fever after pamidronate but no significant toxicity was observed with either treatment. We conclude that both agents are effective in the management of hypercalcaemia of malignancy. At the doses studied, the effects of pamidronate are more complete and longer lasting than those of clodronate.


British Journal of Cancer (1995) 72, 1289-1293

© 1995 Stockton Press All rights reserved 0007-0920/95 $12.00

### A randomised double-blind comparison of intravenous pamidronate and clodronate in the hypercalcaemia of malignancy

OP Purohit[1], CR Radstone[1], C Anthony[1] JA Kanis[2] and RE Coleman[1]

[1]YCRC Department of Clinical Oncology, Weston Park Hospital, Sheffield S10 2SJ, UK; [2]Department of Human Metabolism and Clinical Biochemistry, Royal Hallamshire Hospital, Sheffield S10 2RX, UK.

**Summary** In conjunction with rehydration, the bisphosphonates are the treatment of choice for hypercalcaemia of malignancy. Single infusions of either pamidronate or clodronate are usually effective, but a direct comparison of the two agents given at the highest doses commonly used has not been performed. Forty-one patients (15 breast, 12 squamous carcinomas, four lymphomas, four bladder, two prostate and four others) with hypercalcaemia of malignancy (corrected serum calcium $>2.7\ mmol\ l^{-1}$) persisting after 48 h of saline rehydration were randomly allocated to receive a 4 h intravenous (i.v.) infusion of either pamidronate 90 mg or clodronate 1500 mg. No other systemic anti-cancer treatment was prescribed. There were no significant differences in the post-hydration serum calcium values (mean $3.17\ mmol\ l^{-1}$ for pamidronate and $3.06\ mmol\ l^{-1}$ for clodronate), tumour type or frequency of bone metastases between the two treatments. One patient on each treatment died within 2 days and was not assessable for response. A total of 19/19 (100%) patients achieved normocalcaemia following pamidronate and 16/20 (80%) with clodronate. The median time to achieve normocalcaemia was 4 days (range 2-14) for pamidronate and 3 days (range 2-6) with clodronate. The median duration of normocalcaemia was 28 days (range 10-28 + days) after pamidronate and 14 days after clodronate (range 7-21 days) ($P<0.01$). Two patients who failed to respond to clodronate were successfully treated with pamidronate and achieved normocalcaemia for 14 and >28 days respectively. Two patients experienced fever after pamidronate but no significant toxicity was observed with either treatment. We conclude that both agents are effective in the management of hypercalcaemia of malignancy. At the doses studied, the effects of pamidronate are more complete and longer lasting than those of clodronate.

**Keywords:** hypercalcaemia; pamidronate; clodronate

Hypercalcaemia is a common metabolic complication of malignancy and complicates the clinical course of 5-10% of patients with advanced cancers (Fisken *et al.*, 1980). Intravenous volume expansion to correct dehydration (Hosking *et al.*, 1982), coupled with bisphosphonate therapy to inhibit bone resorption has for some years been the treatment of choice (Ralston *et al.*, 1985; Witte *et al.*, 1987; Singer, 1990). Despite the efficacy of treatment, recurrence of hypercalcaemia occurs unless effective treatment for the underlying cancer is possible. In many situations this cannot be achieved and the median survival for patients with hypercalcaemia of malignancy is less than 3 months (Ralston *et al.*, 1985; Witte *et al.*, 1987; Singer, 1990; O'Rourke *et al.*, 1994).

Three bisphosphonates are currently licensed for the treatment of hypercalcaemia of malignancy in the UK. They are etidronate (Didronel), clodronate (Bonefos, Loron) and pamidronate (Aredia). Both pamidronate and clodronate have been shown to be superior to treatment with etidronate which is the weakest inhibitor of bone resorption (Kanis *et al.*, 1987; Ralston *et al.*, 1989; Gucalp *et al.*, 1992). There has been only one previous randomised comparison of clodronate and pamidronate which showed a more complete and durable control of hypercalcaemia with pamidronate (Ralston *et al.*, 1985). However, the doses of both clodronate and pamidronate were lower than currently recommended. In this study we have performed a randomised comparison of the two agents at the maximum currently recommended dosages (Thiebaud *et al.*, 1988; Nussbaum *et al.*, 1993; O'Rourke *et al.*, 1993). The aim was to identify any difference in duration of normocalcaemia following treatment with clodronate or pamidronate.

### Patients and methods

We studied 41 patients with hypercalcaemia of malignancy associated with various tumour types (Table I). Patients were eligible for treatment when adjusted serum calcium was $>2.7\ mmol\ l^{-1}$, and if they remained hypercalcaemic after 48 h of rehydration with 3 l of normal saline per day. Patients were randomly allocated to receive either pamidronate 90 mg or clodronate 1500 mg, both administered in 500 ml of normal saline over 4 h. All treatments were administered on an inpatient basis and intravenous hydration was continued at $2-3\ l^{-1}\ day^{-1}$ until normocalcaemia was achieved. No other systemic anti-cancer treatment or drugs known to influence bone metabolism were administered during the period of hypercalcaemia and when possible for a total of 28 days after bisphosphonate administration to enable the duration of normocalcaemia to be assessed.

Two days before treatment with the bisphosphonate and on the morning of bisphosphonate treatment and on days 2, 4, 7, 10, 14, 21 and 28 thereafter, blood and urine were collected for analyses. These included measurement in the blood of haemoglobin, platelets and white cells including a differential white cell count. Serum calcium, albumin, phosphate, urea, creatinine, and alkaline phosphatase were measured by Technicon SMAC. Serum calcium was adjusted for fluctuations in albumin concentrations. Measurements of calcium, creatinine and hydroxyproline in urine were performed on the second voided, early morning sample of urine collected over a 2 h period after an overnight fast and acidified before storage at $-20°C$. Calcium and hydroxyproline concentrations were expressed as a ratio of creatinine concentration. Routine observations including temperature were recorded at least twice daily.

The protocol defined retreatment of hypercalcaemia as follows. Those failing to respond to the first bisphosphonate, or developing recurrence of hypercalcaemia within 7 days were to be retreated with the alternative agent, whereas those developing recurrent hypercalcaemia more than 7 days after initial treatment were to be retreated with the same bisphosphonate. Any patient experiencing a third episode of hypercalcaemia was to receive the alternative bisphosphonate. The blind remained unbroken to study investigators and patients

It was not anticipated that a clinically important difference in the frequency of achieving normocalcaemia (adjusted

Correspondence: OP Purohit
Received 9 March 1995; revised 7 June 1995; accepted 9 June 1995

Table I Details of tumour type, treatment and mean post-hydration urinary and calcium serum

| | Pamidronate (n = 20) | | Clodronate (n = 21) | |
|---|---|---|---|---|
| | Bone metastases | | Bone metastases | |
| Tumour type | positive | negative | positive | negative |
| Breast | 5 | – | 10 | – |
| Squamous | 3 | 4 | – | 4 |
| Lymphoma | 1 | 1 | 1 | 2 |
| Bladder | 2 | 1 | 1 | – |
| Prostate | – | – | 2 | – |
| Others | 2 | 1 | 1 | – |
| Mean post-hydration serum calcium (mmol $l^{-1}$) | 3.17 | | 3.06 | |
| Mean post-hydration urinary calcium (mmol $mmol^{-1}$ creatinine) | 1.92 | | 1.77 | |

One patient died in each arm within 2 days of randomisation.

serum calcium <2.65 mmol $l^{-1}$) could be detected and the study size was designed to identify an anticipated doubling of the duration of normocalcaemia from 10 days for clodronate to 20 days for pamidronate. The duration of normocalcaemia was defined as the time from bisphosphonate treatment to recurrence of hypercalcaemia (adjusted serum calcium >2.60 mmol $l^{-1}$). Patients who failed to achieve normocalcaemia were excluded from the duration of normocalcaemia analysis. It was planned to recruit 60 patients to the study to give a 90% chance of detecting this difference but with a planned interim analysis after recruitment of 40 patients. On the basis of the interim analysis showing a difference between treatments which was significant at the $P = 0.01$ level, the study was discontinued and the results are reported here.

Symptomatic response was assessed using two questionnaires. The first recorded the intensity of pain, analgesic consumption and mobility according to the ECOG performance status (WHO, 1979) and the second quality of life (QOL) using the Rotterdam Symptom Checklist (RSCL); (De Haes *et al.*, 1990). This well-validated instrument for measuring QOL in cancer patients comprises physical, functional and psychological components which when combined give a global score of QOL. Both of these questionnaires were completed by the patients before treatment and after 7 and 14 days.

Standard parametric and non-parametric statistical methods were used for analysis of results. These included ANOVA, the Mann–Whitney *U*-test, and paired *t*-test for changes during treatment (Mathews *et al.*, 1990). For the comparison of duration of normocalcaemia a Mann–Whitney life-table analysis was performed. The duration of normocalcaemia was censored if additional anti-cancer treatment was prescribed before the study end at 28 days.

### Results

Of the 41 patients, 20 were allocated to treatment with pamidronate and 21 to clodronate. The two groups were well balanced in terms of post-hydration prebisphosphonate treatment serum calcium, urinary calcium excretion and frequency of bone metastases (Table I).

Two patients died within the first 48 h (one in each treatment group) owing to progressive malignancy and could not be included in the efficacy analysis. Of the 39 evaluable patients, all 19 treated with pamidronate became normocalcaemic compared with 16/20 (80%) patients given clodronate ($P = >0.1$). The median time to normocalcaemia was 3 days for clodronate and 4 days with pamidronate. Figure 1 shows the adjusted serum calcium for the two groups and Figure 2 the actuarial analysis for the duration of normocalcaemia ($n = 19$ for pamidronate and $n = 16$ for clodronate). The number of patients observed at each time point is shown in Figure 2. For clodronate, patients did not reach day 28 because of death due to disease (five patients), too ill to attend (two patients), required systemic treatment (three patients) or had recurrence of hypercalcaemia. For pamidronate the censored patients were death due to disease (five patients), too ill to attend (three patients) or required systemic treatment (four patients). The median duration of normocalcaemia was 14 days (range 7–21 days) following clodronate treatment and 28 days (range 10–>28 days) for pamidronate ($P = 0.01$).

Figure 1 Adjusted serum calcium before and after treatment (mean ± s.e.m.) in 20 patients given pamidronate (solid line) and 21 patients given clodronate (dotted line).

Figures 3a and b show the serial changes in the fasting urinary excretion of calcium and hydroxyproline respectively. Urinary calcium decreased following treatment with both agents but rose again more rapidly following clodronate, with significant differences ($P = <0.01$) appearing between the two groups from 10 days. Changes in hydroxyproline were less marked but similar to the responses in urine calcium in that there was a significant decrease in urinary hydroxyproline in both groups followed by an earlier rebound in the clodronate-treated group.

Figure 4 shows the serial changes in lymphocyte count. Consistent with advanced malignancy many patients had suppressed lymphocyte counts before treatment. There was a small but significant decrease ($P = <0.05$) in lymphocyte count during the first 7 days in the patients given pamidronate, but this was of no clinical significance. There was no significant change in haemoglobin, neutrophils or platelets. The only toxicity observed was fever during the first 24–48 h in three patients after administration of pamidronate, requiring treatment with paracetamol. Patients did not report any other side-effects of treatment. Serum creatinine rose in five patients treated with clodronate. Figure 5 shows the serial changes in serum creatinine. A steady rise was seen (median 166, range 79–581 $\mu$mol $l^{-1}$) in five patients in the clodronate treated group between days 2 and 7. No cause could be found for these changes and they occurred before any recurrence of hypercalcaemia.

The control of hypercalcaemia was associated with an improvement in the pain score in both the treatment groups after 7 days (Figure 6a). This improvement was more marked and statistically significant ($P = <0.05$) in the pamidronate treated group. Because some of the patients studied were confused and drowsy, completion of the QOL questionnaires was incomplete ($n$ = 14 for pamidronate and $n$ = 16 for clod-

**Figure 2** Actuarial plot to show the duration of normocalcaemia following successful treatment of hypercalcaemia in patients given pamidronate ($n$ = 19, -□-) or clodronate ($n$ = 16, -■-).

**Figure 3** (a) Serial changes in urinary excretion of calcium expressed as a molar ratio to creatinine excretion (mean ± s.e.m.). Pamidronate ($n$ = 18, solid line) and clodronate ($n$ = 19, dotted line). (b) Serial changes in urinary excretion of hydroxyproline expressed as a molar ratio to creatinine excretion (mean ± s.e.m.). Pamidronate ($n$ = 18, solid line) and clodronate ($n$ = 19, dotted line).

**Figure 4** Lymphocyte count following treatment (mean ± s.e.m.). Pamidronate ($n$ = 18, solid line) and clodronate ($n$ = 19, dotted line).

**Figure 5** Serum creatinine following treatment (mean ± s.e.m.). Pamidronate ($n = 20$, solid line) and clodronate ($n = 21$, dotted line).

ronate). Nevertheless, the limited data collected showed a significant improvement in QOL following treatment of the hypercalcaemia. However this was not maintained in the clodronate group principally owing to recurrence of hypercalcaemia (Figure 6b).

None of the patients in the pamidronate group showed primary resistance to treatment. Two patients were successfully retreated with pamidronate after recurrence of hypercalcaemia and one failed to respond to retreatment with either pamidronate or clodronate. All the other pamidronate treated patients ($n = 16$) went onto specific anti-cancer treatment and therefore were not assessable for response to retreatment. In the clodronate group, two of the four patients who failed to respond to clodronate were successfully treated with pamidronate with a duration of normocalcaemia of 14 and >28 days. The other two patients were considered too unwell to justify further intervention and died with their hypercalcaemia uncontrolled. Four patients were successfully retreated with clodronate after recurrence of hypercalcaemia and four failed to respond to retreatment. Again, all the other clodronate treated patients ($n = 12$) were given specific anti-cancer treatment and were not assessable for response to retreatment.

### Discussion

Both pamidronate and clodronate were effective in treating hypercalcaemia in this group of patients with advanced malignancy. The small difference in the response rate was not significant although it is of interest that two of three patients with hypercalcaemia resistant to clodronate did respond satisfactorily to pamidronate. Our study confirms the hypothesis that the duration of action of pamidronate is significantly longer than clodronate even when the latter is given at the maximum recommended dose (O'Rourke *et al.*, 1993). These results are consistent with those reported by Ralston *et al.* (1989). In their study, the proportion of patients achieving normocalcaemia on day 6 was 14/16 (87.5%) with pamidronate and 6/16 (37.5%) with clodronate and the duration of normocalcaemia was median 29 (range 18–90) days ($n = 6$) for pamidronate and 12 (range 9–45) days ($n = 7$) for clodronate. The higher response rate in our study is attributable to the higher doses used (Thiebaud *et al.*, 1988; Body *et al.*, 1987; Nussbaum *et al.*, 1993). The observed difference between the two bisphosphonates is due to the potency and different mechanism of action. Clodro-

**Figure 6** (**a**) Change in the pain score following treatment (mean ± s.e.m. expressed as a percentage of the baseline score). Pamidronate ($n = 17$, solid line) and clodronate ($n = 18$ dotted line). (**b**) Quality of life as determined by the RSCL (mean ± s.e.m. expressed as a percentage of the baseline score). Pamidronate ($n = 14$, solid line) and clodronate ($n = 16$, dotted line).

nate is less potent than pamidronate and acts as a direct poison to osteoclasts compared with pamidronate which is not only more potent than clodronate but also inhibits the osteoclastic activity, and in addition has been shown to inhibit the precursor cells to become mature osteoclasts (Fleisch, 1983).

Urinary calcium was suppressed following inhibition of bone resorption to a similar extent with both agents but rose again more rapidly following clodronate. Changes in hydroxyproline were less marked probably owing to the contribution from non-osseous sources of collagen, e.g. breakdown of soft tissue metastases.

Both treatments were well tolerated apart from fever in three patients with pamidronate and an increase in serum creatinine following clodronate in five patients. Fever and lymphopenia are well-recognised side-effects of pamidronate and attributed to cytokine release. The fever is generally mild and transient. It can be treated with paracetamol and becomes less marked if retreatment is required. Lymphopenia is generally transient but in our patients appeared to persist for the duration of observation. The sole unwanted effect of clodronate was the rise in serum creatinine in five patients raising the possibility of treatment-related toxicity, this was unexpected but in none was this attributable to a rise in serum calcium. Renal toxicity has been reported previously with both etidronate and clodronate (Bounameaux *et al.*, 1983) after bolus infusions. However, renal function in patients with osteoporosis and Paget's disease has been unaffected by clodronate, while in hypercalcaemia of malignancy an improvement in renal function has generally been seen. Pamidronate does not appear to have any toxic effects on the kidney at least up to and including the dose we used. A small study of pamidronate given at a dose of 90 mg over 1 h showed no effect on either chromium-labelled EDTA measurement of renal clearance or urinary excretion of *N*-acetylglucosamine (NAG) protein and $\beta_2$-microglobulin (Tyrell *et al.*, 1994). Further studies on the effect of clodronate on renal functions are under evaluation.

In the context of palliation of advanced malignancy, effects on symptoms and quality of life are of paramount importance. The bisphosphonates are known to be useful agents for the treatment of metastatic bone pain (Ernst *et al.*, 1992; Purohit *et al.*, 1994; Tyrrell, 1994). In this study pain improved with both pamidronate and clodronate, although only in the pamidronate-treated group did this reach statistical significance. In this study we attempted to measure quality of life using the Rotterdam Symptom Checklist (RSCL) (De Haes *et al.*, 1990). Because of the poor medical state of many of these patients, data could only be collected on a proportion of the patients studied. However it was clear that control of hypercalcaemia resulted in a reduction in the RSCL score (improvement in quality of life). After 7 days the RSCL scores diverged as patients in the clodronate group began to experience recurrence of hypercalcaemia. These data support, in a more formal way, previous studies indicating that treatment of normocalcaemia is a useful palliative manoeuvre which improves quality of life in terminal cancer patients (Ralston *et al.*, 1990; Ernst *et al.*, 1992; Purohit *et al.*, 1994; Tyrrell, 1994).

We conclude that both bisphosphonates are effective in the management of hypercalcaemia of malignancy. At the doses studied, the effects of pamidronate are more complete and longer lasting than those of clodronate.

**Acknowledgements**

We are most grateful to all the consultants at Weston Park Hospital for referral of patients for this study and to Mrs Di Crowe for secretarial support.

**References**

BODY JJ, POT M, BORKOWSKI A, SCULIER JP AND KLASTERSKY J. (1987). Dose-response study of aminohydroxypropylidine bisphosphonate in tumour-associated hypercalcaemia. *Am. J. Med.*, **82**, 957-963.

BOUNAMEAUX SH, SCHIFFERLI J, MONTANI JP, JUNG A AND CHATELANAT F. (1983). Renal failure associated with intravenous diphosphonates. *Lancet*, **1**, 471.

DE HAES JCM, KNIPPENBERG FCE AND NEIJT JP. (1990). Measuring psychological and physical distress in cancer patients: application of the Rotterdam Symptom Checklist. *Br. J. Cancer*, **62**, 1034.

ERNST DS, MACDONALD N, PATERSON AHG, JENSON J, BRASHER P AND BRUERA E. (1992). A double blind, crossover trial of intravenous clodronate in metastatic bone pain. *J. Pain Symptom Management*, **7**, 4.

FISKEN RA, HEATH DA AND BOLD AM. (1980). Hypercalcaemia - a hospital survey. *Q.J. Med.*, **196**, 405-418.

FLEISCH H. (1983). Bisphosphonates - mechanisms of action and clinical applications. In: Peck WA (ed.). *Bone and Mineral Research*, Vol. 1. pp. 319-357. Excerpta Medica: Amsterdam.

GUCALP R, RITCH P, WEIRNIK PH, SARMA PR, KELLER A, RICHMAN SP, TAUER K, NEIDHART J, MALLETTE LE, SIEGEL R AND VANDEPOL CJ. (1992). Comparative study of pamidronate disodium and etidronate disodium in the treatment of cancer-related hypercalcaemia. *J. Clin. Oncol.*, **10**, 134.

HOSKING DJ, COWLEY A AND BUCKNALL CA. (1982). Rehydration in the treatment of severe hypercalcaemia. *Q.J. Med.*, **200**, 473-481.

KANIS JA, URWIN GH AND GRAY RES. (1987). The effects of intravenous etidronate on skeletal and calcium metabolism. *Am. J. Med.*, **82**, (suppl. 12A) 55.

MATHEWS JNS, ALTMAN DJ, CAMPBELL MJ AND ROYSTON P. (1990). Analysis of serial measurements in medical research. *Br. Med. J.*, **300**, 230.

NUSSBAUM SR, YOUNGER J, VANDEPOL CJ, GAGEL RF, ZUBLER MA, CHAPMAN R, HENDERSON IC AND MALLETTE LE. (1993). Single-dose intravenous therapy with pamidronate for the treatment of hypercalcaemia of malignancy: Comparison of 30, 60-, and 90-mg dosages. *Am. J. Med.*, **95**(3), 297-304.

O'ROURKE NP, McCLOSKEY EV, VASIKARAN S, EYRES K, FERN D AND KANIS JA. (1993). Effective treatment of malignant hypercalcaemia with a single intravenous infusion of clodronate. *Br. J. Cancer*, **67**, 560-563.

O'ROURKE NP, McCLOSKEY EV AND KANIS JA. (1994). Tumour induced Hypercalcaemia: A case for active treatment. *Clin. Oncol.*, **6**, 172-176.

PUROHIT OP, RADSTONE CR, OWEN J, ANTHONY C AND COLEMAN RE. (1994). High dose intravenous pamidronate for metastatic bone pain. *Br. J. Cancer*, **70**, 554-558.

RALSTON SH, GARDNER MD, DRYBURGH FJ, JENKINS AS, COWAN RA AND BOYLE IT. (1985). Comparison of aminohydroxypropylidene diphosphonate, mithramycin, and corticosteroids/calcitonin in the treatment of cancer-associated hypercalcaemia. *Lancet*, **1**, 907-910.

RALSTON SH, GALLAGHER SJ, PATEL U, DRYBURGH FJ, FRASER WD, COWAN RA AND BOYLE IT. (1989). Comparison of three intravenous bisphosphonates in cancer-associated hypercalcaemia. *Lancet*, **2**, 1180-1182.

RALSTON SH, GALLACHER SJ, PATEL U, CAMPBELL J AND BOYLE IT. (1990). Cancer Associated Hypercalcaemia; morbidity and mortality: experience in 126 patients. *Ann. Intern. Med.*, **112**, 499-504.

SINGER FR. (1990). Role of the bisphosphonate etidronate in the therapy of cancer-related hypercalcaemia. *Semin. Oncol.*, **17**, 34.

THIEBAUD D, JAEGER P, JACQUET AF AND BURCKHARDT P. (1988). Dose response in the treatment of hypercalcaemia of malignancy by a single infusion of the bisphosphonate AHPrBP (APD). *J. Clin. Oncol.*, **6**, 762-768.

TYRRELL CJ ON BEHALF OF THE AREDIA MULTINATIONAL COOPERATIVE GROUP. (1994). Role of pamidronate in the management of bone metastases from breast cancer: Results of a non-comparative multicenter phase II trial. *Annals of Oncology*, **5**, 37-40.

TYRRELL CJ, COLLINSON M, MADSEN EL, FORD JM AND COLEMAN T. (1994). Intravenous pamidronate: Infusion rate and safety. *Annals of Oncology*, **5**, 27-29.

WHO. (1979). WHO Handbook for Reporting Results of Cancer Treatment. WHO: Geneva.

WITTE RS, KOELLER J, DAVIS TE, BENSON AB, DURIE BG, LIPTON A, STOCK JL, CITRIN DL AND JACOBS TP. (1987). A randomized study in the treatment of cancer-related hypercalcaemia. *Arch. Intern. Med.*, **147**, 937.